# Tilapia Lake Virus Vaccine Development: A Review on the Recent Advances

**DOI:** 10.3390/vaccines11020251

**Published:** 2023-01-23

**Authors:** Japhette E. Kembou-Ringert, Dieter Steinhagen, John Readman, Janet M. Daly, Mikolaj Adamek

**Affiliations:** 1 Department of Infection, Immunity and Inflammation, Great Ormond Street Institute of Child Health, University College London, 30 Guilford Street, London WC1N 1EH, UK; 2 Fish Disease Research Unit, Institute for Parasitology, University of Veterinary Medicine Hannover, Buenteweg 17, 30559 Hannover, Germany; 3 School of Veterinary Medicine and Science, University of Nottingham, Sutton Bonington LE12 5RD, UK

**Keywords:** tilapia lake virus, fish vaccine, vaccination, vaccines in aquaculture

## Abstract

Tilapia tilapinevirus (or tilapia lake virus, TiLV) is a recently emerging virus associated with a novel disease affecting and decimating tilapia populations around the world. Since its initial identification, TiLV has been reported in 17 countries, often causing mortalities as high as 90% in the affected populations. To date, no therapeutics or commercial vaccines exist for TiLV disease control. Tilapia exposed to TiLV can develop protective immunity, suggesting that vaccination is achievable. Given the important role of vaccination in fish farming, several vaccine strategies are currently being explored and put forward against TiLV but, a comprehensive overview on the efficacy of these platforms is lacking. We here present these approaches in relation with previously developed fish vaccines and discuss their efficacy, vaccine administration routes, and the various factors that can impact vaccine efficacy. The overall recent advances in TiLV vaccine development show different but promising levels of protection. The field is however hampered by the lack of knowledge of the biology of TiLV, notably the function of its genes. Further research and the incorporation of several approaches including prime–boost vaccine regimens, codon optimization, or reverse vaccinology would be beneficial to increase the effectiveness of vaccines targeting TiLV and are further discussed in this review.

## 1. Introduction

Tilapia fish, also known as tilapiine or tilapiine cichlids, are part of a group of three main genera (*Oreochromis*, *Sarotherodon*, and *Tilapia*) within the family *Cichlidae*, which differ from each other by several characteristics notably their reproductive behavior [1]. By being a grazing fish, tilapia play an important role in maintaining the ecological balance of freshwater ecosystems, and they are often used to control algae and mosquito larvae in many aquatic environments. Because they can tolerate high salinity, high water temperature, and low dissolved oxygen, tilapia fish can easily be grown to high densities. Indeed, tilapia aquaculture, mainly of Nile tilapia (*Oreochromis niloticus*), has recently become one of the fastest-growing trades in the aquaculture industry, and the second most cultured species after carps, with an estimated worldwide production of approximately 6.3 million metric tons in 2018 [2], and a global market expected to reach USD 9.2 billion by 2027 [3].

The success of tilapia as a commercial species is partly related to the fact that tilapia fish are overall highly resistant to viral, bacterial, and parasitic diseases when compared with other commonly cultured fish [1]. The few commercially significant tilapia diseases mainly include infections with bacteria such as *Streptococcus* sp. and *Aeromonas* sp. (both accounting for approximately 55% of tilapia infectious diseases) [4], and protozoan diseases such as *Trichodina* (which heavily infest the gills and can sometimes result in high mortality rates in young fish) [5]. Development of antibiotics and vaccines has helped address bacterial infections, whereas protozoan infection treatments involve topical treatments with formalin or with copper sulfate and salt.

Until recently, the most significant viral pathogen of tilapia included an irido-like virus, a large family of double-stranded DNA viruses, which has been credited with massive, synchronized die offs at infected rearing facilities [6], but an intraperitoneally administered vaccine has been developed for this viral infection. Other viral pathogens of tilapia have also been reported, including a herpes-like tilapia larvae encephalitis virus [7] and the viral nervous necrosis (NNV) betanodavirus [8], all accounting for only 22.6% of the common causes of infectious diseases in aquaculture [4].

**Figure 1:**
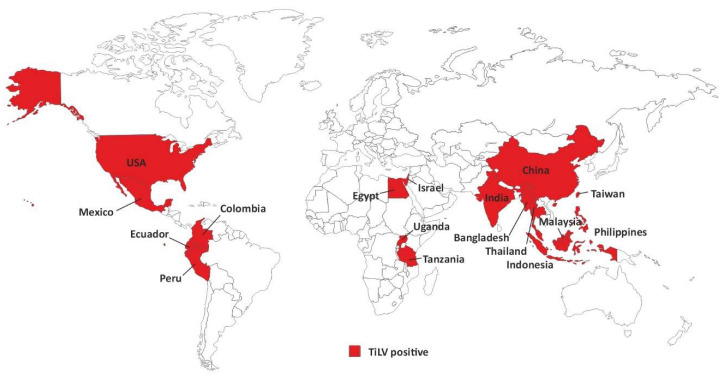
Global presence of TiLV: in red are the countries where the presence of TiLV was reported.

Tilapia lake virus (TiLV) has recently emerged as a main viral pathogen for tilapia aquaculture. TiLV is a causative agent of a lethal novel infectious disease affecting tilapia fish populations around the world, with mortality as high as 90% in some of the affected tilapia fish populations [9]. Since the first reports of the tilapia lake virus disease (TiLVD) in Israel [10], outbreaks of TiLVD have been reported in multiple countries worldwide and the presence of this virus has been overall confirmed in 17 countries, including Ecuador, Colombia, Egypt, Thailand, Taiwan, India, Malaysia, Bangladesh, Uganda, Tanzania, Peru, Mexico, Philippines, Indonesia, the USA, and most recently China [11] (Figure 1), some of which are major producers of tilapia fish species. Thus, a special global alert was issued by the Food and Agriculture Organization of the United Nations (FAO) about TiLV outbreaks and the threat that this virus imposes to tilapia farming and food security worldwide [12].

Moreover, retrospective analyses of TiLV infection in tilapia hatcheries in Thailand from 2012 to 2017 revealed that fries and fingerlings from TiLV-infected hatcheries have been exported worldwide, raising the concern that the virus could have probably been “exported” to over 40 countries around the world [13]. The presence of TiLV in fries and fingerlings demonstrates that all the life stages of tilapia are vulnerable to TiLV infection since vertical transmission of the virus has also been reported [14,15].

The basic reproductive number R0 (number of secondary infections resulting from introducing a single primary infected individual fish into a susceptible host population) for TiLV infection has recently been estimated to be 5.2 [16]. Such a high R0 number implies that the potential for TiLV infection to result in an epidemic (or pandemic) is significant. Furthermore, although the protective humoral response mounted by tilapia after exposure to the virus is relatively long (4 months) [17], this immunity seems to wane over time, allowing subsequent re-infection of individuals becoming susceptible again. Another challenge is the viral persistence in immunologically privileged brain tissue, where the virus can reside for long time after infection and could potentially lead to infection and subsequent new outbreaks in naïve fish. There is therefore an urgent need to include an effective vaccination program among the disease mitigation strategies developed to control the spread of TiLV.

Given that several vaccine development approaches for TiLV infection are currently being explored and suggested, there is a need for a comprehensive overview of the different principles pertaining to these approaches and their implications in vaccine efficacy. In this review, we therefore attempt to analyze these different approaches, while discussing their principles and efficacy as well as the commonly used vaccine administration routes, especially those employed in aquaculture vaccine delivery. The factors that can affect vaccination efficacy of tilapia fish are also discussed together with the major challenges associated with TiLV vaccine development.

## 2. A Brief Overview of Fish Vaccination

A vaccine is a biological substance or preparation used to stimulate the adaptive immunity, induce the activation of cytotoxic T-cells and antibody producing B-cells, and provide immunity against a specific disease or a group of diseases. Vaccines, as biological agents that elicit an immune response to a particular antigen, are often prepared from the causative agent of a disease, from its fragments, or from a synthetic substitute that is acting as an antigen without inducing the disease [18]. The process of vaccinating therefore means that the immune system of the fish is exposed to either the entire pathogen or part of a pathogen prior to infection with a virulent wild-type pathogen.

Since the 1940s, fish vaccination has become the most cost-effective and sustainable method of controlling infectious diseases in aquaculture [19]. It has allowed for the prevention and control of a wide range of bacterial and viral infections. Although antibiotics or chemotherapeutics have been implemented for the treatment of some of these aquatic diseases, drug resistance issues and safety concerns remain major drawbacks for these approaches [20]. Many viral vaccines, either monovalent or multivalent, have been successfully developed and currently over 26 licensed fish vaccines are commercially available worldwide for use in a variety of fish species [21], with few of them, summarized in Table 1, aiming at protecting fish against several families of viruses including rhabdoviruses, birnaviruses, orthomyxoviruses, alphaviruses, alloherpesviruses, and iridoviruses [21].

As previously mentioned, viral vaccines are usually prepared from weakened or inactivated forms of the virus, or one of its surface proteins or viral components. Common types of fish vaccines include whole pathogen vaccines (containing inactivated or attenuated microorganisms), subunit vaccines, virus-like particles (VLPs), and nucleic acids-based vaccines.

Some vaccines produce a poor immunological response on their own and therefore adjuvants are often needed to improve vaccine immunostimulatory capacities. Adjuvants are a group of structurally heterogeneous compounds capable of intrinsically modulating the immunogenicity of an antigen [22]. They are therefore capable of enhancing the immune response and eliciting cytotoxic T lymphocyte responses to generate a heightened local or systemic immune response activation [23].

Standardized in vivo disease challenge models are used for testing the efficacy of vaccines. Bath and co-habitation challenge models closely mimic the natural route of exposure and spreading of the virus, although these models are more difficult to control than injection challenge approaches where each individual fish receives the same dose of the pathogen. When testing vaccine efficacy, pathogen load can be measured by various virus titration methods (plaque assay or 50% tissue culture infective dose—TCID_50_) or viral nucleic acid quantification ((RT)-qPCR), whereas immunological markers of protection can be analyzed by immunoassays to measure pathogen-specific antibody levels (ELISA, SNT). RT-qPCR or RNA-seq can be used to monitor the expression patterns of major immune-related genes allowing us to measure antiviral responses, inflammation, antigen presentation, T- and B-cell responses, etc. The cellular responses (e.g., T-cell and B-cell migration and proliferation) after vaccination can be evaluated by immunohistochemistry and flow cytometry [24]. Assessing survival rates after vaccination and challenge are also critical when evaluating a novel vaccine. This is the reason why the percent of cumulative mortality and the relative percent survival (RPS) are calculated at a specific time and are often used to determine vaccine efficacy [25]. The RPS value is often calculated according to the following formula:

RPS (%) = [1 − (cumulative mortality of the vaccine-treated group/cumulative mortality of the control-treated group)] × 100)

## 3. Types of Fish Vaccines and Current Vaccine Approaches for the Control of TiLV Disease and Infection

Over the years, several types of vaccines have been developed to tackle viral infections of aquatic relevance. These vaccines have been based on several approaches, all aiming at triggering the immune response against a pathogen and evoking the cellular memory response to establish protection against the virus of interest. Although the most popular virus vaccines of aquaculture are based on an inactivated virus or recombinant subunit proteins, novel attenuated and DNA vaccines have also been explored, and are now more and more routinely used in cyprinids and salmonids to address diseases caused by viruses such as koi herpesvirus (KHV), infectious hematopoietic necrosis virus (IHNV), and salmonid alphavirus (SAV) [21].

### 3.1. Inactivated Vaccines

The first produced viral vaccine of fish was an inactivated two-strain vaccine against carp rhabdovirus causing spring viremia of carp [26]. It was based on two inactivated spring viremia of carp virus (SVCV) isolates emulsified in oil and administered by injection. Today, although showing variable efficacy, most virus vaccines used in aquaculture are inactivated vaccines.

Inactivated vaccines consist of viral particles isolated from the diseased fish, cultured, and then treated to eliminate their infectivity potential, as well as their disease-producing capacity, while retaining their immunogenicity. Inactivation can be performed chemically (using inactivating agents such formalin, formaldehyde, ethylamine, or β-propiolactone), physically (using heat), or by ultraviolet (UV) irradiation to reticulate the viral surface glycoproteins interacting with cellular receptors during infection [27]. Because inactivated vaccines are often less efficient at generating protective immunity, immunologic adjuvants and multiple (so-called booster) immunizations might be required to enhance immunity [28]. These types of vaccines are usually administered by intraperitoneal (IP) injection [21,27] and present several advantages including their stability, relative safety, and ease of preparation.

In a recent study conducted by Zeng et al. [29], the efficacy of β-propiolactone (BPL)-inactivated vaccines against isolate TiLV 2017A was tested in tilapia, in the presence and absence of a Montanide IMS 1312 VG adjuvant. The authors found that the highest RPS values (85.7% and 64.3%) were obtained when the inactivated vaccine doses (extrapolated from virus titers before inactivation) were high (1 × 10^8^ TCID_50_/mL and 1 × 10^7^ TCID_50_/mL, respectively) and the adjuvant was present, suggesting that both the amount of inactivated vaccine used, and the presence of an adjuvant can greatly impact the efficacy of inactivated vaccines.

The authors also found that BPL-inactivated vaccines could induce high levels of both TiLV-specific serum IgM and neutralizing antibodies by 3-week post-primary immunization (WPPI), which was even more apparent when virus vaccine doses were 1 × 10^8^ TCID_50_/mL and 1 × 10^7^ TCID_50_/mL in the presence of the adjuvant, with neutralizing antibodies titers reaching significant levels around 6 WPPI. The expression of immune-related genes such as IL-1β, TNF-α, IFN-γ, CD4, and MHC Ia and II were also analyzed, and the authors found that the levels of all these genes were significantly higher in both kidney and spleen of vaccinated fish, especially around 6 WPPI, when inactivated vaccine doses were high and administered together with the adjuvant. This indicates that cellular (MHC-1α and MHC-II) and humoral immunity (CD4) together with the cytokines’ response (TNF-α, IL-1β, and IFN-γ) are all involved in the generation of this specific anti-TiLV response observed in the vaccinated tilapia. Moreover, after being challenged with virulent TiLV 2017A, tilapia vaccinated with BPL-inactivated vaccines showed lower viral loads (especially those vaccinated with higher doses of vaccine in the presence of adjuvant) in liver, spleen, and kidney as opposed to the control, unimmunized fish, demonstrating that vaccination resulted in an inhibition of viral proliferation. Interestingly, the authors found that BPL-inactivated vaccines yielded higher protection rates than formaldehyde-inactivated vaccines, suggesting that the inactivation method could as well have an impact on the efficacy of chemically inactivated vaccines.

In another study by Mai et al. [30], the efficacy of both a water-based heat-inactivated vaccine (HKV) and a formalin-inactivated vaccine (FKV), both administered with no adjuvant, was tested against the TiLV strain TH-2018-K. Similar to Zeng et al. [29], mortality onset in the vaccinated groups, after challenge with a virulent TH-2018-K TiLV strain, was around 7 days post challenge, although Mai et al. [30] used a longer vaccination trial timeline (of 70 days) than Zeng et al. (57 days) [29]. Both the HKV and FKV vaccines were found to confer significant protection to juvenile tilapia, with survival rates of 81.3% and 86.3% and RPS values of 71.3% and 79.6% for HKV and FKV, respectively.

The major differences in protection between these two vaccines were mostly at the level of the immune-related gene expression profiles in the kidney and spleen of tilapia fish. Although the authors found a significant increase in IgM mRNA levels in the head kidney, at 21 days post-vaccination (dpv) for both HKV and FKV vaccines, IgT mRNA levels were nevertheless significantly high at 21 and 28 dpv in the head kidney and the spleen (respectively) only in fish vaccinated with the HKV vaccine. Similarly, CD8 mRNA levels were significantly upregulated at 21 dpv in the spleen of fish vaccinated with the HKV vaccine. 

On the contrary, IgD mRNA levels were significantly high at 21 dpv in the head kidney of fish vaccinated with the FKV vaccine. A significant upregulation of CD4 mRNA levels was also observed in this group of fish at 21 dpv in the head kidney, whereas CD8 levels in this group were also high at 21 dpv although not significantly. Serum IgM levels were also significantly high in both HKV- and FKV-vaccinated groups, especially at 28 dpv. Although the authors did not quantify the levels of secreted IgT antibodies in the mucus, they nevertheless found that mucus Ab IgM levels were also significantly high in the FKV-vaccinated group. These results indicate that both humoral and cell-mediated immune responses are being activated during vaccination with both HKV and FKV. 

In a subsequent study [31], both HKV and FKV vaccines were also employed to investigate if TiLV-specific IgM antibody (anti-TiLV IgM) levels generated in Nile tilapia brood stock could confer passive immunity to juvenile tilapia and if vaccination of parental brood stock would result in protection of larvae via passive maternal antibody transfer. In this study, male and female brood stock were IP immunized with HKV and FKV. This was followed by a booster immunization 3 weeks after primary vaccination. One week after the booster, male and female brood stock initially separated were then allowed to mix and mate. Blood, fertilized eggs, and larvae were then sampled weekly and IgM antibody levels were measured, while sera collected from immunized (with HKV or FKV) female brood stock were used for intramuscular passive immunization of naïve juvenile tilapia.

Like the previous study of Mai et al. [30], anti-TiLV IgM antibodies could also be produced in most of both male and female brood stock vaccinated with either the HKV or FKV; moreover, these antibodies could also be detected in the fertilized eggs and larvae of vaccinated brood stock. Interestingly, higher levels of maternal antibody were observed in fertilized eggs from brood stock vaccinated with HKV than those vaccinated with FKV. Although these anti-TiLV IgM antibodies could be detected in 1–3-day old larvae, they appeared to be of short persistence since they could no longer be detected in 7–14-day old larvae from the vaccinated brood stock. Additionally, antibodies elicited by brood stock vaccination were able to confer 85% (for sera from HKV-vaccinated individuals) to 90% (for sera from FKV-vaccinated individuals) protection to naïve juvenile tilapia against a challenge with a virulent TiLV strain, demonstrating that passive immunization can be achieved with sera from vaccinated individuals. Nevertheless, it would have been interesting to evaluate how long such a passive immunization lasts in the juvenile tilapia, as passive immunization could be another potential strategy for the control of TiLV infection.

The studies on the generation of inactivated TiLV vaccines could benefit from research conducted on other viruses. In aquaculture, multiple inactivated RNA virus vaccines have been tested. A greater potency (81.9% RPS) against viral encephalopathy and retinopathy (VER) disease and high IgM production have also been observed with a formalin-inactivated vaccine, when it was administered by IP injection in European sea bass [32]. Interestingly, in this same study, the BPL-inactivated VER vaccine also induced a relatively high specific IgM production, whereas the IP-injected heat-inactivated vaccine demonstrated a high variability in sera IgM levels, although these levels were high in some individuals. It is also worth noting that formalin-, BPL- and heat-inactivated vaccines that were all administered by immersion in this study yielded very poor and uneven IgM responses among the samples examined by the authors, as opposed to IgM responses in IP-injected vaccinated groups, emphasizing the suitability of IP inoculation as the best administration route for inactivated vaccine formulations. Another remark from this study of Nuñez-Ortiz et al. [32], which might be relevant for future work with TiLV, is the fact that some RNA viruses might be resistant to chemical and physical inactivation, as is the case for some betanodaviruses; proper care might thus be required to verify the complete inactivation prior to vaccination.

### 3.2. Live/Attenuated Viral Vaccines

"Live" attenuated virus (LAV) vaccines are often prepared from virus isolates displaying attenuated virulence or a naturally low virulence toward a target host. Live vaccines can be attenuated by chemical mutagenesis, serial passages in cell culture under abnormal conditions, or by genetic manipulation [21]. Because they remain replication competent, live vaccines tend to be more immunogenic than inactivated preparations. Indeed, this ability to proliferate or enter the host stimulates greater cellular responses both innate and adaptive, which in turn lead to a potent and lasting immune response [33]. Given their usually high efficacy, the use of an adjuvant to enhance the vaccine efficacy of live vaccines is not always required and a single dose might therefore be enough to stimulate a heightened immune response and RPS reaching 100% [34].

Live attenuation by replication in a foreign host leads to a wild-type virus accumulating mutations that adapt it to the foreign host while potentially impairing its virulence in its natural host. It is a long process that can take years or even yield poorly attenuated strains still capable of rapidly reverting to a virulent wild-type genotype after re-introduction in the natural host [35]. This is even more apparent in viruses such as coronaviruses, for instance, which is capable of recombining with other wild-type viruses in nature, resulting in a fully virulent strain [36] that further complicates the development of an attenuated live vaccine against such types of viruses.

The development of the molecular gene manipulation of pathogens has allowed the genetic modification of mutant viruses to generate live vaccine candidates exhibiting attenuated phenotypes. Indeed, the deletion of virulence genes or regulatory genes linked to virulence has been successfully applied to large DNA viruses such as KHV [37], leading to the development of multiple live viral vaccine candidates against KHV of carp currently considered for use in several countries [38].

An attenuated vaccine against TiLV disease, generated by serial passage in cell culture, has been experimentally developed in Israel. This patented vaccine (US20160354458A1) is yet to be commercialized. To determine the number of passages required to obtain attenuated avirulent TiLV vaccine candidates, Bacharach et al. [39] tested passages 12, 17 and 20 of live-attenuated TiLV virus (isolate 4/2011) generated by sequential in vitro passages in a permissive cell line. P12, P17, and P20 vaccine candidates were thus administered by IP injection of PBS containing 1.3 × 10^2^ TCID_50_/mL of TiLV virus to Nile tilapia. Three weeks after vaccination, the fish were challenged through cohabitation with diseased fish (infected by a 6 h cohabitation with diseased fish held in a tank where virulence was increased by 4 passages of wild type TiLV). In this experimental setting, mortalities induced by challenges with the wild-type strain began at days 2 to 4 post-challenge and lasted for 5 to 7 days. Interestingly, only the attenuated TiLV strains induced by 17 and 20 sequential passages (P17 and P20) appeared to be efficient at inducing a considerable protection in the vaccinated fish, yielding RPS values between 56% (for P20) and 58% (for P17) [39].

Despite their high efficacy and long-term protection as well as their ability to elicit both cellular and humoral immunity, live vaccines present major drawbacks, especially related to their safety of use which hinder their extensive use as commercial vaccines. These potential risks, including the risk of reversion to virulence, the display of residual virulence, or the onset of virulence in immunocompromised vaccinates, must first be addressed to ensure their safe and commercial use. This probably explains why Bacharach et al. [39], when evaluating the efficacy of the previously mentioned attenuated TiLV vaccine candidates, also conducted safety studies in which large numbers of fish were injected either with 10-fold doses of the vaccine candidates or with 5 back passages of each variant to address issues such as potential residual virulence and possible reversion to virulence. Although both approaches (5 back passages or a 10-fold increase in vaccine quantity) did not result in the appearance of undesired side effects, the long-term monitoring was not reported, meaning that long-term safety concerns remain to be addressed and assessed. This is especially important when considering that TiLV is a segmented virus, capable of reassortment [40,41], which thus increases the risk of emergence of new viral subtypes. Furthermore, TiLVD has been reported in fish from the natural environment [10,42], where reassortment events cannot easily be monitored and can lead to the development of new pathogenic strains.

Although a highly protective and low pathogenic gene-deleted live attenuated candidate vaccine against infectious spleen and kidney necrosis virus (another viral infection causing high mortality and economic losses to the fish culture industry in Asia) has recently been successfully developed by Zeng et al. [43]. Such a knockout of virulent gene(s) approach for the TiLV vaccine development remains challenged by the lack of knowledge of the function of the different genes encoded by the 10-segment genome of TiLV. The development of a reverse genetics approach such as that of influenza A viruses is thus highly needed as it will certainly be a first step towards TiLV genetic modification to generate gene-deleted attenuated TiLV vaccine candidates.

The detailed vaccination strategies against TiLV for both inactivated and live-attenuated vaccines are summarized in Table 2. 

### 3.3. Subunit/Acellular Vaccines

The development of subunit vaccines harnesses the ability of certain protein antigens to elicit immune reactions that can sometimes lead to high affinity, isotype-switched antibodies. Subunit vaccines indeed consist of only a subset of viral proteins or determinants that are often formulated with purified components of viruses rather than the intact viral particle [21]. Since subunit vaccines cannot replicate in the host, they present no risk of reversion or pathogenicity to the host. Subunit vaccines direct immune responses towards specific viral components or determinants, often those found at the viral envelope, mediating viral binding and virus entry [33].

Protein subunit vaccines are, for instance, generated through the recombinant synthesis of protein antigens using various recombinant expression systems or through protein isolation and purification methods. Although this eliminates the possibility of severe adverse effects, it raises the necessity to have several booster doses as well as the addition of an adjuvant to achieve a stronger and more durable immunization [44]. Virus-like particle (VLP) vaccines, on the other hand, are subunit parts of a virus that self-assemble from viral structural proteins into particles morphologically resembling authentic virions [45]. Unlike the actual virus particles, VLPs lack genomic material (empty virus particles) and only present several copies of the same or few antigens on their surface [33].

These types of vaccines present the advantage that, although composed of viral structural proteins that can be highly immunogenic, they are non-infectious and therefore preclude any possibility of reversion to a pathogenic infectious particle. They are therefore suitable for vaccinating individuals with a compromised immune system. Although several VLP production systems that can allow flexibility and precise tailoring of VLPs exist, their development remains complex, and their assembly is technically challenging, especially for enveloped VLPs [45].

Over the years, few successful subunit vaccines currently used in aquaculture have been developed using *E. coli*-based expression systems. These include an infectious pancreatic necrosis virus VP2 vaccine, a G-glycoprotein subunit vaccine against SVCV, and a VP2/VP3 capsid proteins subunit vaccine against infectious pancreatic necrosis virus [21]. Yeast-based expression systems have also been developed and explored, resulting in the development of a yeast-based subunit vaccine against the infectious salmon anemia virus (ISAV) and HE and F proteins [46]. Other expression systems such as baculovirus-infected insect cells are increasingly being explored in aquaculture to produce subunit vaccines [47,48,49], including a recombinant glycoprotein vaccine against viral hemorrhagic septicemia virus (VHSV) which has been shown to induce neutralizing antibodies in rainbow trout [48]. Given their many benefits and advantages including their suitability for oral administration, plant-based platforms for subunit vaccine production are also being recently introduced in aquaculture [50]. Although no plant-produced fish vaccine has been commercialized to date, few studies have nevertheless explored their potential in aquaculture vaccine production. This is, for instance, the case for a recently developed plant-made nervous necrosis VLP vaccine against Atlantic cod nervous necrosis virus [51], and a plant-produced subunit vaccine candidate against a piscine myocarditis virus in Atlantic salmon *Salmo salar* [52], with the nervous necrosis VLP vaccine yielding RPS values ranging from 63.6 to 86.5% and an increase in mRNA expression levels of genes encoding for RIG-1 and STAT1 observed in the heart and spleen of salmon fish immunized with the subunit VLP vaccine against piscine myocarditis virus. VLP-based vaccines that have been successfully licensed and commercialized to date are those targeting viruses of human importance. These include the Engerix-B^®^ (manufactured by GlaxoSmithKline Biologicals S.A.) VLP vaccine against Hepatitis B virus produced in yeast [53], the Hecolin1 VLP vaccine against Hepatitis E virus produced in *E. coli* [54], and the Gardasi and Cervarix^®^ (manufactured by GlaxoSmithKline Biologicals S.A.) vaccines, both targeting human papillomavirus and produced in yeast and insect, respectively [55,56]. 

Candidate subunit vaccines against TiLV were also tested recently with encouraging results. A VP20 subunit vaccine (deriving from the gene product of TiLV segment 8) expressed in *E. coli* was recently developed and evaluated by Zeng et al. [57] against TiLV. When IM was injected in tilapia, this recombinant rVP20 protein induced significantly high antibody titers as early as two weeks after immunization as well as a significant increase in the expression of immune-related genes encoding for MHC-Ia, MHC-II, IL-1β, TNFα, and CD4, indicating that cellular immune responses were induced. Although this subunit rVP20 vaccine was administered with an adjuvant M402-enhanced aluminum, it could only confer a partial protection of tilapia against a challenge with TiLV isolate A2017 since an RPS value and a survival rate of only 51.3% and 52.5%, respectively, were obtained. To improve the potency and efficacy of this VP20-based vaccine, Zeng et al. [57] explored a DNA prime–protein boost regimen incorporating both a VP20-plasmid-based DNA vaccine (pV-optiVP20) for prime immunization and the rVP20 for boosting. A vaccination regimen which significantly increased antibody response, virus neutralization, and vaccine protection is discussed in Section 6.

In another study by Chamtim et al. [58], recombinant proteins deriving from TiLV segment 9 (Tis9) and segment 10 (Tis10) and expressed in and purified from *E. coli* were also evaluated for their efficacy at protecting juvenile hybrid red tilapia. These potential subunit vaccines were administered by IP injection with a Montanide ISA 763 adjuvant 4 weeks before an IP challenge with a wild-type TiLV virus. Vaccination with Tis9 yielded cumulative mortality and RPS values of 43.33 ± 5.77% and 27.78 ± 9.62% at 28 days post TiLV challenge, respectively, whereas in fish vaccinated with Tis10 cumulative mortality and RPS values were 33.33 ± 15.28% and 44.45 ± 25.46% respectively. When Tis9 and Tis10 were combined and administered concomitantly, RPS value increased to 55.56 ± 9.62%; a value close to that was obtained after vaccination with rVP20 protein alone. This suggests that these two proteins might not be strong immunogens (although in silico analyses of both proteins revealed several putative linear and conformational B-cell epitopes in their coil structures) when compared with VP20 deriving from TiLV segment 8. It is also interesting to observe in this study that Tis10 vaccine (and the pcDNA-Tis10 DNA vaccine) conferred a better protection than the Tis9 vaccine (and pcDNA-Tis9 DNA vaccine), reinforcing the notion that not all viral proteins are suitable immunogens. Although cumbersome, identifying viral proteins capable of eliciting strong immunogenicity when used as subunit vaccine remains of great importance if one is to achieve effective vaccine protection.

TiLV proteins deriving from segment 5 (S5) and segment 6 (S6) were also successfully expressed in *E. coli* and purified as soluble and insoluble protein subunits [59]. When used as antigens for the immunization of Nile tilapia, both proteins were able to elicit a significant anti-TiLV antibody production, suggesting that these two proteins, similarly to the one deriving from segment 8, are immunogenic, and could thus serve as potential vaccine candidates. Generating subunit vaccines based on the immune recognition of these two proteins is of relevance in the context of TiLV infection, as these two proteins have been previously predicted to possess signal peptides [60], which strongly suggests that they are likely part of the virus envelope.

Recently, an in vivo cloning approach using the yeast *Saccharomyces cerevisiae* for recombinant protein expression was successfully applied for the expression of recombinant TiLV open reading frames (ORFs) [61]. Such a system could allow for the efficient production of a large amount of recombinant viral proteins that can serve vaccine purposes. Although they are yet to be licensed for use in aquaculture, yeast-based vaccines are becoming of increasing interest in aquaculture, and the strategy described by Cueva et al. [61] is particularly interesting as it allows for the cloning of one or more viral fragments in a single plasmid; therefore, it opens avenues for the production of VLP vaccines, which to the best of the authors’ knowledge, are yet to be developed for TiLV disease and infection control.

Bañuelos-Hernández et al. [62] have also proposed to produce a recombinant oral vaccine in microalgae using viral vectors. This suggested approach is based on the development of a geminivirus viral vector that will allow the cloning of two or three TiLV genes in a single vector (multigene viral vector) that will then be used for microalgae transformation. This recombinant protein production strategy in microalgae is a promising avenue when considering that antigen bio-encapsulation in algae cells could significantly improve antigen preservation in the fish gastrointestinal tract [63], leading to an increase in vaccine absorption in the small intestine and a better vaccine bioavailability. Moreover, when considering that tilapia naturally feed on vegetable detritus and microalgae, recombinant subunit antigen production in microalgae could represent an easier and adequate antigen production strategy for vaccines, especially in tilapia aquaculture.

In light of the above-discussed advances, it is evident that the development of subunit vaccines (based on recombinant protein expression) for the control of TiLVD is already well established. Cloning and expression strategies in yeast, such as the one described by Cueva et al. [61], will also certainly allow the generation of new subunit vaccine platforms such as VLP vaccines and subviral particles (SVPs) containing viral surface antigens (glycoproteins) that can act as immunogens [45]. Although this notion is greatly challenged by the lack of knowledge as to which proteins serve as the viral glycoproteins of TiLV, the identification of the envelope proteins of TiLV and their expression and assembly into VLP particles closely resembling a native virus would greatly enhance the immunogenicity of such vaccine platforms and elicit higher antibody and immune responses. This was previously demonstrated for VLP vaccines developed against betanodavirus, such as the nervous necrosis virus (NNV) [51,64,65].

### 3.4. Nucleic Acid Vaccines

Nucleic acid vaccines are simple particle vaccines consisting of DNA or mRNA encoding the antigen(s) of interest. These vaccine types are relatively simple to generate since they only require a genetic sequence encoding for the viral antigen of interest and a delivery platform [66]. Moreover, their safety of administration (no possibility of reversion to a pathogenic state) and fast-track design make them ideal vaccine platforms that can be deployed rapidly and easily in the case of disease outbreaks [35]. Nucleic acid vaccines have also been demonstrated to be potent inducers of both humoral and cellular adaptive immune responses [67,68], and although the use of DNA or mRNA as the genetic material dictates the production pipeline, the stability, and the storage conditions of these vaccines, their production remains relatively simple, flexible, and scalable.

#### 3.4.1. DNA Vaccines

DNA vaccines consist of an expression plasmid carrying the gene encoding the vaccine antigen. This gene of interest is flanked by promoter and termination elements that facilitate its expression within eukaryotic cells. When expressed by cells in the vaccinated host, it elicits an immune response which eventually leads to protection against the pathogen from which the antigen derives [69].

**Figure 2:**
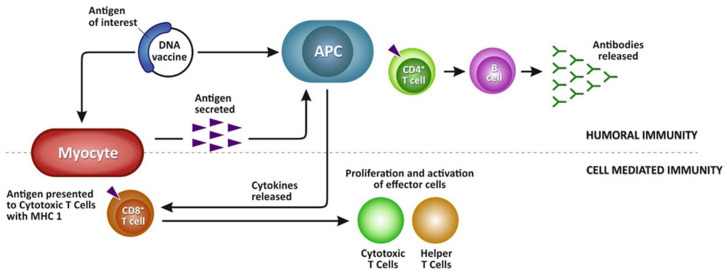
Humoral and cellular immune mechanisms after vaccination with a DNA vaccine. Following administration of a DNA vaccine bearing the antigen gene of interest, cells of the host take up the vaccine and utilize their cellular machinery to produce and express the foreign protein deriving from this antigen of interest. The secreted or presented antigen is then recognized by the immune cells such as antigen-presenting cells, which in turn leads to the activation of both humoral and cellular defense mechanisms.

DNA vaccines are usually administered intramuscularly; however, intraperitoneal delivery of DNA vaccines is also feasible but it appears to require considerably higher amounts of DNA than IM delivery [70]. Following administration of a DNA vaccine, cells of the host take up the vaccine and utilize their cellular machinery to produce and express the foreign protein deriving from the antigen of interest. This in turn leads to antigen recognition by immune cells such as antigen-presenting cells (APC) and activation of both humoral and cellular defense mechanisms (depicted in Figure 2) [71,72]. Since this foreign antigen is produced inside the vaccinated host organism via genetic expression, the duration of the immune response is relatively long lasting, especially against viruses, as has been demonstrated for IHNV and for VHSV [21,69,71]. Despite their safety of use, DNA vaccines often still require adjuvants for their administration since one technical hurdle pertaining to this type of vaccines is their inefficient uptake by the host cells [73]. DNA vaccines can also be constructed to be multivalent, meaning to provide immunity and protection against multiple antigens, using genes coding for multiple antigens when designing the plasmid for antigen expression.

Over the years, several DNA vaccines have been experimentally tested in various fish species and some of them, including a DNA vaccine against rhabdovirus diseases, have demonstrated promising results. Few DNA vaccines have been licensed for use in aquaculture, including a DNA vaccine against IHNV commercialized in Canada and a DNA vaccine against a salmonid alphavirus subtype 3 (pancreas disease virus) marketed in the European Union [21].

A VP20 (deriving from segment 8) plasmid DNA vaccine (pV-optiVP20) has been developed and evaluated against TiLV by Zeng et al. [57]. IM vaccination of tilapia with this codon-optimized vaccine vector resulted in a significant increase in antigen-specific serum IgM which was reinforced after booster immunization 3 WPPI. The pV-optiVP20-DNA vaccine also induced high expression levels of immune-related genes such as IL-1β, TNF-α, CD4, and MHC-II demonstrating that both humoral and cellular immune responses were activated. IgM levels in the spleen were also significantly up-regulated and a significant decrease in viral loads was observed in the liver and kidneys after being challenged with a virulent TiLV 2017A strain, although vaccination with this DNA vaccine resulted in only a 50% survival rate and a 48.7% RPS value in the PV-optiVP20-vaccinated fish group.

Another DNA vaccine (pcDNA3.1–ORF10) against TiLV infection was developed and tested by Yu et al. [74] for its efficacy against TiLV in Nile tilapia. After IM vaccination with this DNA vaccine, the authors observed a significant upregulation of immune-related genes encoding for IgM, TLR2, MyD88, IL8, TNFα, IFN-γ, and NF-κB in the spleen, liver, and kidney of pcDNA3.1–ORF10-vaccinated tilapia, especially those encoding for IFN-γ in the liver, IgM, IFN-γ, and NF-κB in the spleen, and IL-8 and TNF-α in the kidneys. A delayed onset of mortality was also observed in the vaccinated group, with RPS values increasing from 60.71% to 85.72% as the amount of DNA vaccine used was increased. Significantly lower viral loads were also observed in the spleen, liver, and kidneys of the pcDNA3.1–ORF10-vaccinated and challenged tilapias fish, overall demonstrating that pcDNA3.1–ORF10 can induce protective immunity in vaccinated Nile tilapia.

Chamtim et al. [58] also recently evaluated the efficacy of two candidate DNA vaccines (pcDNA-Tis9 and pcDNA-Tis10) deriving from TiLV segments 9 and 10 by IM injection of juvenile hybrid red tilapia. Interestingly, immunization with these candidate vaccines resulted in RPS values of 50.00 ± 16.67% for pcDNA-Tis10 and 38.89 ± 9.62% for pcDNA-Tis9 using only 5 μg of vector for each candidate vaccine, confirming the highly immunogenic nature of the ORF deriving from TiLV segment 10, over the one deriving from TiLV segment 9.

The initial steps towards the formulation of another DNA vaccine against TiLV, based on a recombinant vector containing the gene deriving from TiLV segment 4, have also been described by Criollo-Joaquin et al. [75]. This amplicon gene could be detected as early as 8 h post-immunization following two injections to juvenile tilapia. It is important to note here that the development of this DNA vaccine formulation was based on the previous hypothesis of Criollo-Joaquin et al. [75] that this gene segment (TiLV segment 4) was encoding for the viral neuraminidase protein of TiLV, which for influenza viruses, is a membrane-bound surface glycoprotein, thus representing a potential gene segment amenable to vaccine development. This hypothesis was later dismissed as Abu Rass et al. [76] demonstrated that this gene segment encodes for the nucleoprotein of TiLV.

Higher levels of protection as well as robust antibody response and successful vaccination were also observed when the surface glycoproteins of VHSV and IHNV were used as antigens in the DNA vaccine expression plasmid [71,77,78].

#### 3.4.2. RNA-Based Vaccines

Like DNA vaccines, RNA-based vaccines are safe since they are non-infectious. Moreover, they are also potent stimulators of immunity [79] and have shown a lot of success over recent years, especially for coronaviruses such as severe acute respiratory syndrome coronavirus 2 [35]. With the exception that the antigen of interest is encoded by an mRNA molecule (mRNA) and that this mRNA needs to reach cytoplasmic or endoplasmic reticulum ribosomes to be translated into protein, the delivery of mRNA vaccines follows quite the same concept as DNA vaccines. However, mRNA molecules are significantly more unstable than DNA and therefore require specialized encapsulation methods and very low temperatures (between −70 °C and −20 °C) for long-term storage, making their distribution difficult in some settings.

The development of mRNA-based vaccines, until recently, was challenged by several factors inherent to the mRNA molecule itself. Indeed, naked mRNA is prone to nuclease digestion. Moreover, it is too large and highly negatively charged to passively cross the cell membrane. Its dense negative charge can electrostatically repulse the anionic cell membrane, and thus greatly reduce its cellular uptake rate, which has been shown to be less than 1 in 10,000 molecules [80]. Efficient delivery systems are therefore crucial to enhance their uptake and facilitate their utilization as vaccine platforms.

mRNA-based vaccines are generally classified as either nonreplicating or self-replicating (self-amplifying). Nonreplicating mRNA constructs tend to have a small size because they lack additional encoded proteins such as the ones encoding for the RNA-dependent RNA polymerase (RDRP) complex [81]. The mRNA sequence encoding for the immunogen of interest is flanked by 5′ and 3′ untranslated regions (UTRs) and harbors both a 5′ cap structure consisting of 7-methylguanosine (m7G) connected by a triphosphate bridge to the first nucleotide and a 3′-poly(A) tail [81]. These structures (5′ cap and 3′-poly(A)) act in preventing the recognition by the cytoplasmic RNA helicases retinoic-acid-inducible gene I (RIG-I) in suppressing 5′–3′ exonuclease-mediated degradation, in recruiting translation initiation factors, and in promoting efficient translation. Self-replicating mRNA constructs on the other hand, encode an RDRP complex necessary for self-amplification, in addition to the components found in nonreplicating constructs [82]. The most currently used self-replicating mRNA vaccines are based on an alphavirus-based vector, in which the RDRP complex derives from alphaviruses such as the Semliki Forest virus, and equine encephalitis viruses [83]. The self-replication feature of these types of mRNA vaccines greatly increases the magnitude and the duration of the expression of the immunogen and enables multiple antigens to be produced from an extremely small dose of vaccine. These vaccine types can therefore induce robust immune responses with the additional advantage that multiple gene sequences can be incorporated into the same replicon to allow the expression of additional immunomodulatory molecules capable of enhancing vaccine potency [84]; although the production and stability of larger replicons are more challenging.

To date, very few mRNA-based vaccines have been developed and reported for use in aquaculture. A replicon mRNA vaccine against ISAV, constructed based on the salmonid alphavirus 3 (SAV3) genome [85], has nevertheless been previously tested in Atlantic salmon by Wolf et al. [86] where IM injection without adjuvant provided high protection against ISAV, although IP administration of this same replicon vaccine did not induce protection [87].

An mRNA-based vaccine platform against TiLV remains to be developed. Given that the alphavirus replicase functions in a broad range of host cells including fish, this field could see significant advances if, for instance, the genes for the structural proteins of the alphavirus were replaced with those of TiLV. Alternatively, the previously developed SAV3-based replicon against ISAV (an orthomyxovirus which is in the same viral order as TiLV) could offer the backbone frame that could allow the development of a self-amplifying mRNA vaccine that could potentially protect tilapia against TiLVD. Table 3 summarizes the different recombinant protein subunits and DNA vaccine strategies currently reported against TiLVD.

### 3.5. Nanoparticle-Based Vaccines

The recent advancement in nanotechnology has facilitated the development of another group of vaccines in which nanoscale-sized particulates termed nanoparticles (or nanocarriers) are used for either virus antigen encapsulation or antigen conjugation. The resulting nanoparticle formulation then serves as an efficient vaccine delivery system that protects the encapsulated antigen from degradation and improves its overall stability as well as vaccine efficacy.

Different nanoparticles have been used in fish vaccine delivery, including nanoliposomes, calcium phosphate, biodegradable polymers, carbon nanotubes, and immunostimulating complexes (ISCOMs) such as poly (lactic-co-glycolic acid or PLGA) and chitosan, the most studied and used form of nanoparticles to date. An interesting feature of nanoparticles is their ability to encapsulate (or to be conjugated to) several types of antigen formulations including inactivated, subunits, and nucleic acids as they mostly serve as delivery systems. In addition to their delivery functions, some nanocarriers also display intrinsic adjuvant properties and are thus capable of activating immune cells [88]. In fact, all nanoparticles can stimulate antibody responses as well as the production of cytokines [89]. Moreover, the encapsulated cargo can also be formulated to include adjuvants and immune stimulatory molecules which can significantly improve antigen immunogenicity [90,91].

Physiochemical properties of nanovaccines such as their surface charge, their size, and their shape, can affect their interactions with APCs and innate immune cells. Although nanoparticles can enter target cells through cellular endocytosis [92,93], due to their very small size, cellular uptake and inflammatory responses have been shown to be affected by the above-mentioned physiochemical properties.

In fact, the size of nanoparticles has been shown to be a key factor in determining the efficiency and mode of cellular uptake. For instance, nanoparticles ranging from 20 to 200 nm in diameter would be readily endocytosed by lymph-node-resident dendritic cells, whereas nanoparticles ranging from 500 to 1000 nm would be mostly taken up by migratory dendritic cells [94]. Nanoparticles with an average size of 200 nm are internalized via clathrin or caveolin-mediated endocytosis, whereas nanoparticles with an average size larger than 500 µm are taken up by micropinocytosis or phagocytosis [95,96]. It was reported that large-sized nanoparticles tend to elicit humoral immune responses, whereas smaller-sized nanoparticles tend to promote cellular-mediated immune responses.

Indeed, it has been previously demonstrated that polymeric PLGA nanoparticles of various sizes (200 nm, 500 nm, and 1000 nm) encapsulating BSA stimulated different immune responses. PLGA nanoparticles with a size of 1000 nm elicited a greater IgG response than that of the 200 nm and 500 nm nanoparticles [97]. This is a differential immune response attributed to the fact that smaller nanoparticles were being more efficiently taken up by the APCs than larger nanoparticles which would instead adhere to the surface of the APCs where they would activate a B-cell response [97,98].

Over the years, several nanovaccine formulations in which the antigen is encapsulated, surface exposed, or recombinantly ensembled (VLPs), have been developed against fish diseases. A nanovaccine in which PLGA nanoparticle carriers were used to encapsulate a DNA vaccine was, for instance, developed against lymphocystis disease virus in Japanese flounder (*Paralichthys olivaceus*) [99,100]. Similarly, PLGA nanoparticles were used to encapsulate a DNA vaccine against IHNV in rainbow trout (*Oncorhynchus mykiss*) [101]. Liposome-formulated nanovaccines against KHV have also been developed including liposome-nanovaccine formulations entrapping formalin-inactivated KHV antigens within the liposomal membrane compartment and used for oral vaccination of common carp (*Cyprinus carpio*) [102,103]. Chitosan nanoparticles have also been extensively studied and widely used in aquaculture for the development of fish vaccines including a chitosan nanoparticle-based vaccine loaded with both an inactivated virus vaccine against ISAV and a DNA coding for the replicase of the alphavirus and serving as the adjuvant [104], as well as a chitosan oral nanoparticle-based DNA vaccine against turbot reddish body iridovirus (a piscine iridovirus) [105].

A chitosan nanoparticle-based vaccine was also recently developed against tilapia lake virus [106]. This immersion vaccine encapsulates a formalin-inactivated TiLV virus (TiLV strain VET-KUTV08) and possesses gill mucoadhesive properties. In a cohabitation challenge model, fish vaccinated with this CN-KV nanovaccine demonstrated better RPS values (68.17%) than those vaccinated with the naked inactivated virus vaccine (25.01%). Moreover, CN-KV-vaccinated fish exhibited higher TiLV-specific antibody response at 14 days post-challenge than their control counterparts (which received either the naked inactivated virus or the chitosan nanoparticles only). Furthermore, under field farming conditions where natural exposure to TiLV was allowed 28 days post-vaccination, the RPS values of CN-KV-vaccinated fish were 52.22% compared with the control group that received chitosan nanoparticles only. Similar to the orally administered PLGA and liposome-formulated nanovaccines, this CN-KV chitosan nanovaccine formulation is of special interest as it allows for less labor-intensive mass vaccination by immersion, thus making its application relevant to farm conditions.

Another biomimetic nanodelivery system (Cs-pS2@M-M) using a DSPE-PEG-Man mannose modified from teleost erythrocyte membranes was also recently used as a delivery carrier for an anti-TiLV DNA vaccine deriving from TiLV segment 2 [107]. Unlike the above-mentioned CN-KV chitosan-based nanovaccine, this biomimetic nanovaccine was administered intramuscularly where it induced a sustained and efficient expression of the plasmid DNA in muscle and spleen tissue. Additionally, this nanovaccine induced a high and effective serum antibody production as well as upregulated expression of some immune-related genes compared with the same dose (10 μg/g) of non-mannosylated nanoparticles or naked DNA vaccine. Indeed, the mannose-vaccinated group demonstrated 76.9% RPS values as opposed to 50.0% RPS values observed in the naked DNA-vaccinated group. Although the intramuscular administration of this vaccine could explain its high efficacy, the use of teleost erythrocyte membranes for nanoparticle coating also provides an efficient immune-induction strategy for vaccination. Indeed, nucleated erythrocyte from teleost fish have recently gained attention as they have been found to play an important role in modulating host immune responses as well as in being associated with antiviral responses against infection [108,109]. Moreover, modifying nanoparticle-coating membranes to specifically target APCs as performed by Gong et al. [107] can significantly increase antigen delivery to APCs and thus effectively enhance T-cell responses [110]. However, the intramuscular administration of this biomimetic nanovaccine remains time-consuming, labor-intensive, costly, and hardly allows for simultaneous mass vaccination in farming conditions.

In addition to chitosan-based and biomimetic membrane erythrocyte-based nanovaccine formulations successfully developed against TiLV (the detailed vaccination strategies for these nanovaccine formulations are summarized in Table 4), the recent and successful use of polyanhydride nanoparticles (biodegradable polymers) for the encapsulation and release of vaccine antigens, such as the RNAi-based virus-specific dsRNA antigen vaccine recently developed against white spot syndrome virus in shrimp [111], for instance, altogether certainly opens exciting avenues for safer and effective vaccine delivery systems readily applicable in aquaculture nanovaccine development. 

Overall, the recent TiLV vaccine developments seem to exhibit different levels yet promising degrees of protection. The identification of immunogenic gene segments such as segments 8 and 10 certainly offer initial effective control measures based on immunization using these segments as antigens.

## 4. Vaccine Delivery Routes 

Fish vaccines are commonly administered by oral, injection (intraperitoneal or intramuscular), and immersion routes. Several factors dictate the choice of the delivery method and these usually include the vaccine type, the fish life stage (larvae, fry, juvenile, adults, or spawning), the vaccine production techniques and its underlying principles, the immunological memory status, the pathogen, and its life cycle (natural infection route), as well as the labor costs [27]. The choice of the delivery route can also significantly influence the immunological response and the protection levels obtained following vaccination [87,112].

### 4.1. Oral Vaccination

This delivery route often involves incorporating the vaccine into fish feed, resulting in the requirement of a large quantity of antigen, even though the level of protection achieved is generally weak and of short duration. Although oral vaccine administration remains simple, safe, easy, less stressful for the fish, and suitable for all fish life stages and sizes, immunological responses obtained via this route of administration are often quite inconsistent and poor. This is probably a result of antigen degradation in the gut, poor antigen transfer rate from fish intestinal lumen to immune reactive cells [113], as well as hypo-responsiveness to a fed antigen (oral tolerance) [114]. This probably explains why antigens delivered via the oral route often require specific encapsulation techniques such as microalgae, nanoparticles, and biofilms, which altogether greatly increase the overall cost of oral vaccine delivery.

In principle, whole pathogen antigens, subunit antigens, and nucleic acids antigens, especially DNA-based vaccines, could all be administered orally [90]. In fact, most nanovaccine formulations reported in aquaculture are administered orally [99,101,102,103,104,105,111]. Nevertheless, vaccine formulation to protect the antigen from stomach degradation while improving the stimulation of protective immunity of oral vaccines is certainly one of the biggest challenges in the development of effective oral vaccines. Yeast cell systems to produce subunit vaccines destined for oral administration have gained ground over recent years, appearing as very good vehicles for oral antigen delivery, which could also serve as potential adjuvants [115]. A few commercial oral vaccines have been produced using these expression systems [116,117] and, as previously mentioned, *Saccharomyces cerevisiae* was recently used to efficiently in vivo clone viral cDNA deriving from the 10 segments of TiLV [61]; thus, offering avenues for improvement of tilapia oral vaccine efficacy.

While vaccination via oral mucosal surfaces seems to provide poor local and systemic immune responses, the exact mechanisms of immune induction (local and systemic) and protection following oral vaccination are yet to be elucidated, especially in tilapia. Furthermore, it will be important to explore and understand if vaccines delivered at other mucosal surfaces, for instance nasal (for nebulized DNA vaccines, for instance) and gills, could elicit better local and systemic responses, although the latter mucosal route might be proven more stressful for the fish than the oral route.

### 4.2. Vaccination by Injection

As opposed to oral vaccination, vaccine administration by injection (IP or IM) requires a lower amount of antigen. Moreover, every fish effectively receives the correct and same dose of vaccine. Vaccination by IP or IM injection is more prolonged when compared with the immersion delivery route [118], but despite these advantages, antigen delivery via injection is labor intensive, restricted to fish of a certain size and life stage, and appears to be more stressful for the vaccinated fish.

DNA vaccines are often administered via an IM route [119], whereas adjuvants, especially oil adjuvants, are often used when IP injection is performed [27]. IP injection seems to be the most common route of administration of inactivated vaccines, as great potency has been observed when major inactivated vaccines such as the formalin-treated vaccine against viral encephalopathy in European sea bass [32], and the UV-inactivated vaccine against genotype red grouper nervous necrosis virus strain It/411/96 [120], were administered by IP injection. The currently reported inactivated TiLV vaccines are equally all administered via IP route [29,30], as well as the recently reported anti-TiLV mannose functionalized biomimetic nanovaccine using modified tilapia erythrocyte membranes [108]. Given that the stress caused by vaccination by injection sometimes leads to mortality, multiple administration of injectable vaccines throughout the fish production cycle cannot be performed, which is a major drawback of injectable vaccines.

Other injection delivery routes such as particle-mediated epidermal delivery by gene gun has been documented for some DNA vaccines, in which DNA vaccines are coated with small gold particles and delivered intradermally by air-pressure [67]. Although this novel technology has been proven effective in fish [121,122], it remains too expensive and thus not cost effective for commercial aquaculture. Interestingly, DNA vaccines can also be delivered intradermally whereby a short electric pulse would then be generated to optimize their uptake by cutaneous antigen-presenting cells (APCs). Subcutaneous administration of DNA vaccine is also feasible, in which case the uptake is conducted by fibroblast and keratinocyte. Transdermal administration, which primarily engages tissue-resident Langerhans cells, as well as intravenous injection (in which the DNA vaccine systematically reaches secondary lymphatic organs), are also possible injection routes for DNA vaccine delivery [33], even though most of these routes are currently not used in aquaculture.

### 4.3. Vaccination by Immersion (Dipping or Bathing)

As opposed to injection, vaccination by immersion is a very simple strategy. Here, fishes are either immersed for about 30 s in a high concentration of vaccine solution (dipping) or immersed for a longer period (several hours) in a lower concentration of vaccine to then be released into culture tanks [123]. The fact that immersion mimics the natural course of infection in fish, probably explains why this approach has commonly been used for live attenuated and vector vaccines [124], although nanovaccines such as the chitosan-based nanovaccine (CN-KV) developed against TiLV [106] can also be administered by immersion. Furthermore, vaccination by immersion can be carried out for fishes of all sizes and life stages; it is less stressful, convenient, rapid, and cost effective as simultaneous immunization of a large number of fish is possible. Despite these many advantages, the immunity via this delivery route seems to take longer to establish (between 3 to 12 months) [125] and seems to be shorter in duration [123], thus requiring additional booster doses.

Vaccine delivery has always been an important issue in commercial aquaculture. There is thus a clear need for the development of mass immunization methods yielding optimal effectiveness with the least amount of stress for the vaccinated fish. Various administration routes have also therefore been investigated and these include ultrasound using DNA-coated microspheres and DNA formulated in liposomes [126,127,128]; even though none of these alternatives has yet provided comparable efficacy to that of IM and IP injections. 

## 5. Factors That Can Influence Vaccine Efficacy

### 5.1. Vaccine Formulation—Dose, Use of Adjuvant, Administration Route, and Addition of a Nanocarrier Delivery System

As previously observed [29,74], vaccine efficacy is ultimately influenced by the dose of the vaccine being administered and the use of adjuvants. This is also exemplified in a study by Alberer et al. [129] in which the efficacy of vaccination against rabies virus with an mRNA vaccine was found to be highly dependent on the dose and the route of administration.

Despite such correlation, an increase in vaccine dose must be taken with cautious considerations given the multiple side effects associated with vaccination, especially with adjuvanted vaccines. Indeed, adjuvanted vaccines may cause inflammation, as well as intra-abdominal lesions such as granulomatous peritonitis, which may be restricted to the abdominal cavity or may also be generalized (resulting in heavy melanisation), leading to a retarded growth [130] and even mortality. These lesions have been found to have a higher incidence in vaccinated fish smaller than 75 g [131]. In addition, vaccination may result in impaired feeding patterns characterized by a prolonged period before a return to normal feeding [132], which may result in growth impairment characterized by a drop in weight and size and a high potential of downgrading of the final fish product.

The vaccine formulation, the use of adjuvant, the route of administration, and the water temperature, as well as the age and body weight of the vaccinated fish, are therefore all factors that can have a significant impact on the efficacy of a given vaccine [133]. It has been shown that a high dose of vaccine combined with the maintenance of the water temperature within a proper range can induce increased immune protection [134,135]. In Atlantic lumpfish (*Cyclopterus lumpus*), for instance, vaccination at a low temperature has been shown to lead to low antibody response against *Aeromonas salmonicida* [134].

In addition to acting as nanocarrier delivery systems, which can potentially protect vaccine formulations from degradation thus increasing their half-lives, nanoparticles can also act as immunostimulatory adjuvants to induce and enhance protective immunity [136]. Moreover, modifying these nanocarriers to present erythrocyte membranes or mannose as ligands on their surface can significantly increase the targeted delivery of vaccine formulations to APCs and thus effectively enhance T cell responses and improve vaccine efficacy. This has been carried out by Gong et al. [107] resulting in a 26.9% increase in vaccine efficacy (RPS) in the group of tilapia fish vaccinated with a DNA vaccine encapsulated in a modified mannose biomimetic nanoparticle incorporating both chitosan and teleost erythrocyte membranes (Cs-pS2@M-M). In fact, vaccine efficacy gradually increased as the DNA vaccine formulation was either naked (50.0%), encapsulated with chitosan nanoparticles (53.8%), then coated with erythrocyte membranes (61.5%), and finally coated with DSPE-PEG-Man-modified mannose (76.9%). Incorporation of nanoparticles in vaccine formulations thus presents the opportunity that it could enhance antigen uptake by targeting specific immune cells and improve vaccine efficacy.

### 5.2. Vaccine Regimen—Heterologous Prime–Boost Regimen 

For most vaccines to induce long-lasting protective immunity, multiple immunizations or boosters might be required to improve their efficacy. Repetitive immunogenic stimulations using different antigen delivery systems to elicit different types of immune responses can increase the intensity and durability of the adaptive immunity. This strategy known as the "heterologous prime–boost" or "mix-and-match" strategy consists of combining different vaccine types targeting the same antigen during the prime and boost phases of vaccination. It has been used by Zeng et al. [57] to improve vaccine efficacy in tilapia challenged with a virulent TiLV strain [57]. In this study, a pVoptiVP20 DNA vaccine was used as the primer vaccine which was then followed by a booster with a rVP20 subunit vaccine 3 weeks after prime vaccination, resulting in a significantly higher survival rate of 72.5% in tilapia inoculated with this approach as opposed to 50% and 52.5% survival rates in fish immunized with either the pV-optiVP20 or the rVP20 alone, respectively. Protein vaccines tend to establish long-term memory by eliciting B-cell responses as well as specific immunogenic T-cell and B-cell epitopes, whereas DNA vaccines induce a full spectrum of immune responses that include cytolytic T cells, T helper cells, and antibodies. The sequential administration of vaccines evoking cellular and humoral immunity via different mechanisms could be more beneficial than that of a single vaccine and could very likely result in higher vaccine efficacy and increased protection, as equally demonstrated in several other studies [137,138,139]. Nevertheless, the boosting regimen, the order of vector injection, and the spacing between the vaccines are all factors that can significantly influence the outcome of the prime–boost immunization regimens [140]. A better understanding of the principles of memory development of B, T, and innate cells, especially in tilapia, will therefore certainly be important for the optimization of prime–boost strategies. It will also be important to define and determine which vaccine is best to use first and second in a regimen, as well as how long the delay should be between immunizations.

### 5.3. Codon Optimization for DNA Vaccines

Of note, the segment 8 gene of TiLV that encodes the VP20 protein serving as antigen in this study by Zeng et al. [57] was codon-optimized for fish usage in a pVAX1 vector. Codon optimization aims at improving protein expression while reducing sequence complexity in a recombinant gene without changing the amino acid sequence. This technique takes advantage of the fact that some amino acids are encoded by more than one codon and that different organisms exhibit bias towards the use of certain codons (preferential usage) over others for the same amino acid [141]. Therefore, if a human gene is to be expressed in *E. coli*, for instance, choosing codons preferentially used by the bacterium can increase the success of protein expression [142]. This approach has been found to significantly increase recombinant protein expression, especially in heterologous systems, and has found potential applications in vaccine development, especially in recombinant protein production [143], to increase the efficiency of gene expression in DNA vaccines [144]. Therefore, it appears as a potential strategy that could greatly increase DNA vaccine efficacy by improving the expression and delivery of DNA vaccine antigens [145].

## 6. Challenges

**Figure 3:**
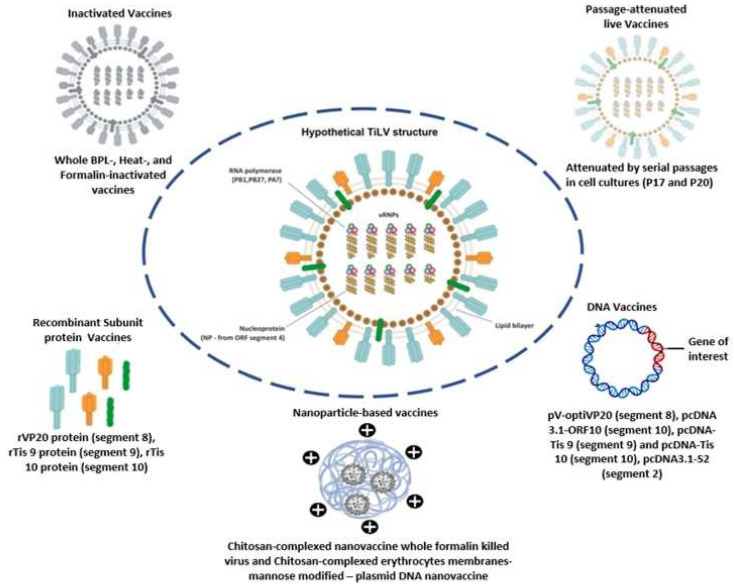
The overall recent vaccine formulations reported against TiLV. A hypothetical structure of TiLV showing the 10 viral RNPs, the lipid bilayer, the PB1 hypothetically complexed with other proteins possibly acting as PA and PB2, and the nucleoprotein deriving from segment 4. The five main vaccine types that have been developed and reported against TiLV are also represented.

Given that cost effectiveness is an essential goal in commercial fish production, it is very likely that, due to their ease of production, safety, and efficacy, DNA vaccines (especially the ones based on the TiLV segment 8 gene product) are the way forward to achieving fish protection by immunization at a large commercial scale. Major challenges to this, however, are the legislative and regulatory requirements pertaining to these vaccines [146] since the cost of licensing could greatly inhibit the use of these types of vaccines in commercial tilapia farming. Nevertheless, the current advances in TiLV vaccine development in general (depicted in Figure 3) certainly allow large-scale tilapia protection with the various vaccine formulations currently reported. Hopefully, these formulations will also be affordable enough to suit the low-scale producers with low-income margins who currently account for most tilapia producers in some countries.

The identification of additional immunogenic gene segments such as segments 8 and 10 should also prompt further research work, as these could inspire the development of highly potent gene-based vaccine vectors with higher efficacy. The fact that the envelope glycoproteins of TiLV are yet to be identified hinders the design of an effective vaccine targeting these specific viral proteins. This is of special importance when considering that: i) similarly to influenza viruses, TiLV is a segmented virus, and as such, antigenic shift (reassortment) can occur during TiLV infection [40]. In fact, it even plays a crucial role in the evolution [41] and dynamics of TiLV infection. Therefore, such a reassortment may have an impact on the efficacy of a proposed vaccine. ii) The design of a vaccine eliciting neutralizing antibodies against such glycoproteins will not just disrupt the life cycle of the virus by preventing its attachment, binding, and internalization, but could also provide sterilizing protection against TiLV infection. In this respect, live attenuated whole virus formulations could be a better option as they present on their surface these virus glycoproteins in their native conformation, and they could elicit neutralizing antibodies recognizing these antigen epitopes. Nonetheless, risks related to their use (risk of virulence reversion and residual virulence) would first need to be addressed.

## 7. Perspectives

The current limited knowledge of tilapia immune response pathways has limited the development and implementation of novel vaccine delivery routes. Furthermore, innate immune mechanisms in tilapia are poorly understood, even though the innate immune system certainly plays a very important role in the protection against infectious diseases. Therefore, more research needs to be conducted to understand this immune branch in tilapia fish as this could reveal the existence of specific immune stimulants (such as short DNA fragments—CpGDNA) that could serve as effective and potent adjuvants presenting fewer side effects, as previously demonstrated by Jørgensen et al. [147].

The understanding of innate antiviral responses in tilapia could equally open perspectives on novel avenues of inducing early protection via trained immunity, whereby some antigens stimulate strong innate antiviral responses that induce early protection against heterologous viruses. This has been demonstrated for several antigenic stimuli including a salmonid rhabdovirus glycoprotein DNA vaccine which induces early protection against heterologous viruses such as Atlantic halibut nodavirus in turbot [148]. Such a strategy could be beneficial as fish vaccines with established safety profiles already exist and could be repurposed as trained immunity-based vaccines for TiLV disease containment.

Given the lack of knowledge of the cellular receptor and envelope glycoprotein(s) of TiLV, the future development of a multi-epitope subunit vaccine is hard to predict. The successful in vivo cloning of all TiLV gene segments in yeast [61] for recombinant protein expression nevertheless offers a promising platform that could lead to the design and generation of safe and potent multiepitope VLP vaccines against this virus. The design of unique multiepitope subunit vaccines against TiLV could take advantage of the current availability of the whole genome sequences of this virus to identify highly immunogenic antigens (or epitopes) in silico that could then be cloned, library expressed, and screened for their ability at inducing a strong immune response in mice, for instance. This reverse vaccinology approach also presents the advantage that previously unknown antigens (such as virulence factors) and even surface-located proteins can be identified, thus making it possible to study the function of some of these antigens, leading to a better understanding of the biology of the pathogen [149].

Although VLP vaccines containing surface glycoproteins can exhibit increased immunogenicity and efficacy, the highly mutational nature of these proteins, well exemplified by the HA and NA of influenza viruses, may require periodic updates of the vaccine antigens. Given the similar nature of TiLV to influenza viruses (segmented RNA virus), a possible solution could be the development of subunit vaccines based on conserved antigen epitopes. Although identification of such epitopes may be cumbersome, this approach could nevertheless be very effective against both newly emerging and previously circulating TiLV strains.

## 8. Conclusions

The emergence of TiLV as a lethal virus in tilapia aquaculture certainly necessitates novel disease prevention and treatment measures. In this regard, vaccines are considered the most sustainable and cost-effective way to address diseases of aquaculture importance and decrease the economic losses due to fish disease outbreaks. The ideal fish vaccine will thus be one that is safe for the animal and the environment, easy to administer, economical for any scale of production, and capable of inducing a strong and protective immunity with minimal possible side effects.

Several vaccine strategies are currently being suggested to control TiLV infection, and although these various strategies are demonstrating promising results in providing immunity against TiLV infection, they still do not rely on the mounting of an immune response against the viral envelope glycoprotein(s) of TiLV (except for the inactivated or live-attenuated vaccine candidates). This is in part because these viral proteins remain to be identified. Nevertheless, given the high potential of TiLV infection resulting in mass mortality, it is essential to further explore other fast-track vaccine development platforms such as multiepitope VLPs and mRNA nanoparticle vaccines. Without doubt, the growing knowledge of the immune pathways of fish will certainly have an impact on the development of novel vaccines and vaccine strategies relying on potent natural adjuvant-like innate immune stimulants, such as the previously identified CpG DNA fragments, which present fewer side effects. As tilapia aquaculture continues to expand globally, novel emerging diseases of aquaculture relevancy will probably arise, and there will certainly be a need for new vaccines to be developed, or old ones to be repurposed. The application of all available biotechnology towards solving these emerging diseases will thus be critical.

## Figures and Tables

**Table 1:** Examples of licensed fish viral vaccines for a variety of fish species.

Pathogen	Virus Family	Major Fish Host	Disease	Vaccine Type	Antigens/Targets
Infectious salmon anemia virus (ISAV)	Orthomyxoviridae	Atlantic salmon	Infectious salmon anemia	Inactivated	Inactivated ISAV
Koi herpesvirus (KHV)	Alloherpesviridae	Carps	Koi herpesvirus disease	Attenuated	Attenuated KHV
Salmonid alphavirus (SAV)	Togaviridae	Salmonids	Pancreatic disease of salmonids	Inactivated	Inactivated SAV
				DNA	Structural polyprotein C-E3-E2-6K-E2
Infectious hematopoietic necrosis virus (IHNV)	Rhabdoviridae	Salmonids	Infectious hematopoietic necrosis	DNA	G-glycoprotein
Spring viremia carp virus (SVCV) Rhabdovirus	Rhabdoviridae	Carps	Spring viremia of carp	Subunit	G-glycoprotein
				Inactivated	Inactivated SVCV
Infectious pancreatic necrosis virus (IPNV) Birnavirus	Birnaviridae	Salmonids, sea bass, sea bream, turbot, Pacific cod	Infectious pancreatic necrosis	Inactivated	Inactivated IPNV
				Subunit	VP2 and VP3 Capsid Proteins
				Subunit	VP2 protein

**Table 2:** Detailed inactivated and passage-attenuated vaccine strategies reported against TiLV infection.

Vaccine Types	Inactivated Vaccines			Passage-Attenuated Live Vaccines	
Vaccine Formulation	BPL-Inactivated [29]	Heat-Killed [30]	Formalin-Killed [30]	P17 [39]	P20 [39]
**Adjuvant**	Montanide IMS 1312 VG	-	-	-	-
**Vaccine dose**	1.8 × 10^8^ TCID_50_/mL	1.8 × 10^6^ TCID_50_/mL	1.8 × 10^6^ TCID_50_/mL	1.3 × 10^2^ TCID_50_/mL	1.3 × 10^2^ TCID_50_/mL
**Number of fish vaccinated**	50 fish/experimental group	25 fish/experimental group	25 fish/experimental group	30 fish/experimental group	30 fish/experimental group
**Immunization regimen**	2 doses–3 weeks apart	2 doses–3 weeks apart	2 doses–3 weeks apart	1 dose	1 dose
**Number of fish challenged**	30 fish/experimental group	16 fish (×2 replicates)	15 (×2 replicates)	30 fish (challenge done by cohabitation with diseased fish)	30 fish (challenge done by cohabitation with diseased fish)
**Vaccine efficacy (RPS %)**	85.7%	71.3%	79.6%	58%	56%
**Survival rate (%)**	86.7%	81.3%	86.3%	62%	64%
**Antibody response**	Serum anti-TiLV IgM reported and neutralizing antibodies detected	Upregulation of IgM, IgT and IgD reported	Upregulation of IgM, IgT and IgD reported	Not reported	Not reported
**T-cell response**	CD4 cell activation reported and significant increase in IL-1β, TNFα, IFN-γ, MHC-II and MHC-Ia	CD4 cell activation (in the kidney) CD8 cell activation (in the spleen)	CD4 cell activation (in the kidney) CD8 cell activation (in the kidney and spleen)	Not reported	Not reported

**Table 3:** Detailed recombinant protein subunit and DNA vaccine strategies reported against TiLV infection.

Vaccine Types	Recombinant Protein Subunit Vaccines			DNA Vaccines			
Vaccine Formulation	rVP20 Protein [57]	rTIS 9 [58]	rTIS10 [58]	pV-optiVP20 [57]	pcDNA 3.1-ORF10 [75]	pcDNA-Tis 9 [58]	pcDNA-Tis 10 [58]
**Adjuvant**	M402 Enhanced aluminium	Montanide ISA 763 adjuvant	Montanide ISA 763 adjuvant	-	-	-	-
**Vaccine dose**	400 µg	200 µg	200 µg	50 µg	45 µg	5 µg	5 µg
**Number of fish vaccinated**	100 fish/experimental group	75 fish/experimental group	75 fish/experimental group	100 fish/experimental group	30 fish/experimental group	75 fish/experimental group	75 fish/experimental group
**Immunization regimen**	2 doses–3 weeks apart	1 dose	1 dose	2 doses–3 weeks apart	2 doses–2 weeks apart	1 dose	1 dose
**Number of fish challenged**	40 fish/experimental group	10 fish (×3 replicates–challenged 4 weeks post vaccination)	10 fish (×3 replicates–challenged 4 weeks post vaccination)	40 fish/experimental group	30 fish/experimental group	10 fish (×3 replicates–challenged 4 weeks post vaccination)	10 fish (×3 replicates–challenged 4 weeks post vaccination)
**Vaccine efficacy** **(RPS %)**	51.3%	27.8 ± 9.6%	44.4 ± 25.4%	48.7%	85.7%	38.9 ± 9.6%	50.00% ± 16.7
**Survival rate (%)**	52.5%	-	-	50%	-	-	-
**Antibody response**	Serum anti-TiLV IgM reported, and neutralizing antibodies detected	Relative increase in sera antibody response (assessed by dot blot assay)	Relative increase in sera antibody response (assessed by dot blot assay)	Increased serum anti-TiLV IgM antibodies and increased serum neutralizing antibodies	Significant upregulation of TLR2, MyD88, IL8, TNFα, INF-γ, and NF- κB	Relative increase in sera antibody response (assessed by dot blot)	Relative increase in sera antibody response (assessed by dot blot)
**T-cell response**	CD4 cell activation reported and upregulation of IL-1β, TNFα, MHC-II and MHC-Ia	Not reported	Not reported	CD4 cell activation reported and upregulation of IL-1β, TNFα, MHC-II and MHC-Ia	Not reported	Not reported	Not reported

**Table 4:** Detailed nanovaccine strategies reported against TiLV infection.

Vaccine Type	Nanoparticle-Based Vaccines	
Vaccine Formulation	Chitosan-Formalin Inactivated TiLV-Complexed Nanovaccine (CN-KV) [107]	Biomimetic Mannose Modified Erythrocyte Membrane—DNA TiLV Segment 2 Nanovaccine (Cs-pS2@M-M) [108]
**Adjuvant**	-	-
**Vaccine dose**	10^3^ TCID_50_/mL	10 μg
**Number of fish vaccinated**	60 fish/experimental group	60 fish/experimental group
**Immunization regimen**	1 dose	1 dose
**Number of fish challenged**	10 fish I.P. challenged and reared in cohabitation with vaccinated fish (in a 1:3 ratio)	33 fish I.P. challenged
**Vaccine efficacy** **(RPS %)**	68.2%	76.9%
**Survival rate (%)**	-	-
**Antibody response**	Increased TiLV-specific serum antibody response—only at 14 dpc (assessed by indirect enzyme-linked immunosorbent assay—ELISA)	High TiLV-specific serum antibody response (assessed by indirect enzyme-linked immunosorbent assay—ELISA). Significant upregulation of IgM
**T-cell response**	Not reported	Significant upregulation of IFN-γ, TNF-α, IL-8, MHC-Iα and CC2

## Data Availability

Not applicable.
